# Whey protein and creatine supplementation in autistic adult women: a call for sex- and age-specific research

**DOI:** 10.3389/fnut.2026.1902181

**Published:** 2026-09-09

**Authors:** Maria Lima Braga de Jesus

**Affiliations:** Independent Researcher, Salvador, Bahia, Brazil

**Keywords:** adult women, autism spectrum disorder, creatine, nutritional supplementation, research priorities, whey protein

## Introduction

Autism spectrum disorder (ASD) is a lifelong neurodevelopmental condition affecting approximately 1%−2% of the global population (1). Despite significant growth in ASD research, a persistent blind spot remains: the nutritional needs of autistic adult women. The overwhelming majority of clinical and nutritional studies have focused on male children, leaving adult women—a population only recently gaining diagnostic visibility—largely unstudied (2).

Late diagnosis is disproportionately common among autistic women, who frequently engage in social masking that delays clinical identification until adulthood (3). A systematic review covering 2013–2024 identified only 43 full-text articles on diet and nutritional status in autistic adults, with virtually none disaggregating findings by sex (2). Crucially, women with higher autistic trait scores consume significantly less protein, fat, vitamins, and minerals than women with lower autistic trait scores (4), creating a distinct risk profile for nutritional deficiencies. This underrepresentation reflects two compounding factors: a historical recruitment bias driven by higher male diagnostic rates and the empirically documented disproportionate exclusion of autistic females from research through standard diagnostic instruments—what has been described as a “leaky” recruitment-to-research pipeline (5, 6).

Against this backdrop, two supplements with mechanistically plausible benefits in ASD have attracted scientific interest: whey protein and creatine. This article argues that the existing evidence base—though derived almost entirely from pediatric and male samples—is sufficient to justify and urgently demand well-designed clinical trials in autistic adult women.

## Whey protein: mechanistic hypotheses

The primary hypothesis supporting whey protein in ASD involves the glutathione (GSH) pathway. Autistic individuals present with markers of elevated oxidative stress, mitochondrial dysfunction, and genetic abnormalities in GSH-related pathways (7). Whey protein is a dairy-derived food ingredient—a complex mixture of proteins including beta-lactoglobulin, alpha-lactalbumin, and immunoglobulins—distinct in origin and biochemical nature from endogenously synthesized compounds such as creatine.

Cysteine-rich whey protein isolates are direct precursors to GSH synthesis, and supplementation has been proposed to restore antioxidant capacity and improve neurobehavioral outcomes (8). Doses studied in the literature range from 20 to 40 g/day of high-quality isolate, with particular attention to cysteine content in the context of the GSH hypothesis. A 90-day randomized controlled trial in preschool-aged autistic children confirmed increases in intracellular GSH, though behavioral improvements were mixed and largely non-significant (8).

Importantly, an *in vivo* spectroscopy study in autistic adults found no significant difference in frontal or occipital brain GSH levels compared with neurotypical controls (9), suggesting that the GSH hypothesis, while robust in pediatric ASD, remains largely unconfirmed or underexplored in autistic adults *in vivo*, underscoring the need for population-specific research. A second relevant pathway is the gut–brain axis: in adults, whey protein supplementation produces measurable shifts in gut microbiota composition, including increases in beneficial bacterial populations (10); these effects are particularly relevant given that food selectivity in autistic women is associated with reduced microbiome diversity (11). Animal model data further support this pathway, demonstrating that whey protein supplementation in a valproic acid-induced ASD model increased beneficial gut bacteria and improved memory performance (12). A third, more modest hypothesis is simple nutritional adequacy: Given systematically lower protein intakes in women with high autistic trait scores (4), supplementation may act primarily by correcting a dietary deficiency rather than via pharmacological mechanisms.

Taken together, these three mechanistic pathways are not mutually exclusive and may operate simultaneously in the same individual. At the cellular level, cysteine derived from whey protein hydrolysis enters the gamma-glutamylcysteine synthetase pathway, driving GSH synthesis in neurons and astrocytes—a process that is ATP-dependent and therefore sensitive to the same mitochondrial dysfunction implicated in ASD (7, 13). At the intestinal level, whey protein peptides—particularly beta-lactoglobulin-derived fragments—act as prebiotics, selectively promoting the growth of *Lactobacillus* and *Bifidobacterium* species and increasing short-chain fatty acid (SCFA) production, which, in turn, modulates enteroendocrine signaling and vagal afferent pathways to the brain (10, 11). At the systemic level, correcting protein insufficiency in autistic women with restricted dietary patterns may improve nitrogen balance, support lean mass maintenance, and reduce fatigue—outcomes with direct relevance to daily functioning and quality of life (4). The relative contribution of each pathway in a given individual will likely depend on baseline nutritional status, microbiota composition, and oxidative stress profile, reinforcing the case for stratified trial designs rather than a one-size-fits-all supplementation approach.

## Creatine: brain energy metabolism and sex-specific biology

In contrast to whey protein, creatine is a naturally occurring nitrogenous compound synthesized endogenously from arginine and glycine, and it is also obtained from dietary animal sources. Its primary mechanism of action is cellular energy buffering through the phosphocreatine (PCr)-ATP system, with a well-established safety profile. Creatine represents a particularly compelling candidate in autistic adult women. ASD is characterized by a biochemical endophenotype of insufficient mitochondrial energy production, with documented impairments in electron transport chain function and ATP metabolism (13). Creatine and phosphocreatine (PCr) constitute the primary cytosolic ATP buffer, and a systematic review and meta-analysis of *in vivo* magnetic resonance spectroscopy studies has documented alterations in creatine and energy metabolism metabolites across multiple brain regions in autistic individuals (14). A 2024 meta-analysis of 16 randomized controlled trials demonstrated that creatine monohydrate supplementation (typically 3–5 g/day) significantly improved memory and information processing speed in adults; however, the authors noted that its impact on attention scores was not significant when considered as a whole (15)—domains centrally impaired in autistic adults.

The case for creatine is further strengthened by *sex-specific biology*. Women have endogenous creatine stores approximately 70%−80% lower than men and lower dietary intake of creatine overall (16). Preclinical data show antidepressant-like effects of creatine exclusively in female rodents—attributed to the high density of estrogen receptors on mitochondria and estrogen's role in mitochondrial bioenergetics (17). Clinically, creatine augmentation in adolescent and adult women with treatment-resistant depression increased brain PCr concentrations and correlated with mood improvement (18). As depression and anxiety are highly prevalent and frequently underdiagnosed comorbidities in autistic women, this psychoneurological dimension adds considerable clinical urgency.

## Barriers and conditions for refutation

Scientific rigor requires equal attention to conditions that would refute these hypotheses. For whey protein, sensory hypersensitivity to textures, tastes, and odors—common in autistic individuals—may render supplementation practically infeasible regardless of biochemical rationale. Adherence failure driven by sensory aversion would constitute *de facto* refutation of efficacy. It should also be noted that, as a dairy-derived product, whey protein may be poorly tolerated by a subset of autistic individuals with sensitivity to milk proteins, which further limits its universal applicability.

For creatine, the critical contraindication is creatine transporter deficiency (CTD), caused by mutations in the SLC6A8 gene (X-linked), which renders oral supplementation ineffective because creatine cannot enter the brain (19). Any clinical trial in this population should include screening for this variant. Furthermore, both hypotheses remain limited by the near-total absence of data on how hormonal fluctuations across the menstrual cycle, perimenopause, or hormonal therapy interact with creatine kinetics or GSH metabolism in autistic women.

## Discussion

The evidence reviewed in this study does not yet permit clinical recommendations for whey protein or creatine supplementation in autistic adult women. What it does support, unequivocally, is the need for rigorously designed trials targeting this population. The mechanistic plausibility of both supplements—grounded in established pathways of oxidative stress, mitochondrial energy metabolism, and sex-specific creatine biology—provides a scientifically sound foundation for such an agenda.

The current literature presents two compounding gaps: the near-absence of nutritional research in autistic adults of any sex and the systematic exclusion of women from existing studies. This exclusion reflects, first, a recruitment bias: because ASD has historically been diagnosed more frequently in males, research samples have been predominantly male by default. Second, it reflects the empirically documented effect of standard diagnostic instruments—such as the autism diagnostic observation Schedule (ADOS)—which disproportionately exclude autistic females from research eligibility, creating a structural barrier to their inclusion that operates prior to and independently of researcher intent (5, 6). In the context of ASD nutritional research, these compounding biases are particularly consequential given the documented differences in dietary patterns, body composition, hormonal milieu, and the female autistic phenotype itself.

The author of this article has personal experience with both supplements, shared here transparently as informal observational experience and not as clinical evidence. Whey protein isolate was used for approximately 9 months in 2020–2021 to complement protein intake and support muscle mass in association with functional training, with a modest positive outcome and no sensory or digestive difficulties noted. Creatine monohydrate was used for approximately 6 months in association with resistance training; no effects on cognition, energy, or mood were observed, though a small increase in muscular strength was noted. A transient increase in intestinal gas during the first week resolved with increased hydration and dietary fiber. These observations are shared transparently as lived experience, not as clinical evidence, and are consistent with the broader literature: effects appear to be context-dependent, ergogenic rather than cognitive in the absence of a confirmed deficit, and highly individual. They also illustrate a practical consideration: sensory acceptability and gastrointestinal tolerance—factors rarely addressed in supplementation trials—may be determinants of real-world adherence in autistic women.

The question of which autistic women might benefit most from each supplement is clinically central. For creatine, the most promising candidates are those with confirmed mitochondrial dysfunction, elevated fatigue, executive function deficits, or comorbid depression, and the critical exclusion criterion is the presence of CTD (SLC6A8 variants), which renders oral supplementation ineffective. For whey protein, women with documented protein deficiency, restricted dietary patterns, and evidence of oxidative stress or gut microbiota dysbiosis represent the subgroup most likely to benefit. Future trials should therefore be designed around biological stratification—mitochondrial function, GSH status, hormonal profile, dietary assessment, sensory processing measures, and relevant genotyping—rather than broad diagnostic categories alone.

Future trials should incorporate, as minimum requirements: stratification by biological sex and hormonal status; assessment of baseline oxidative stress markers and mitochondrial function; brain creatine quantification via proton magnetic resonance spectroscopy; genotyping for creatine metabolism variants (*GATM, GAMT*, and *SLC6A8*); validated measures of sensory food processing as determinants of adherence; and outcome domains relevant to autistic women's daily functioning, including cognitive flexibility, executive function, interoception, and quality of life.

It is equally important to acknowledge what remains unknown. The GSH hypothesis, while robust in pediatric ASD, remains largely unconfirmed or underexplored in autistic adults *in vivo*. The gut microbiome data linking whey protein to autism-relevant outcomes derive primarily from animal models, with human data limited to general adult populations. The sex-specific antidepressant effects of creatine, though compelling, have not been tested in autistic women specifically. These are not reasons to abandon these hypotheses—they are precisely the reasons to test them.

The cost of continued inaction falls disproportionately on a population that has already waited too long for science to recognize its specific needs. Autistic adult women deserve nutritional research designed around them, not extrapolated from populations that do not represent them.
